# High-Intensity Interval Training vs. Medium-Intensity Continuous Training in Cardiac Rehabilitation Programs: A Narrative Review

**DOI:** 10.3390/medicina60111875

**Published:** 2024-11-15

**Authors:** Alexandru Dan Costache, Alexandra Maștaleru, Maria Magdalena Leon, Mihai Roca, Radu Sebastian Gavril, Diana Elena Cosău, Andreea Rotundu, Alice Ioana Amagdalinei, Ovidiu Mitu, Irina Iuliana Costache Enache, Florin Mitu

**Affiliations:** 1 Clinical Rehabilitation Hospital, 700661 Iasi, Romania; adcostache@yahoo.com (A.D.C.); leon_mariamagdalena@yahoo.com (M.M.L.); roca2m@yahoo.com (M.R.); rgavril87@yahoo.com (R.S.G.); e_cosau@yahoo.com (D.E.C.); andreea.rotundu@gmail.com (A.R.); amagdalinei_alice_95@yahoo.com (A.I.A.); mitu.florin@yahoo.com (F.M.); 2 Faculty of Medicine, “Grigore T. Popa” University of Medicine and Pharmacy, 700115 Iasi, Romania; mituovidiu@yahoo.co.uk (O.M.); ii.costache@yahoo.com (I.I.C.E.); 3 “St. Spiridon” Emergency County Hospital, 700111 Iasi, Romania; 4 Romanian Academy of Medical Sciences, 927180 Bucharest, Romania; 5 Romanian Academy of Scientists, 050044 Bucharest, Romania

**Keywords:** cardiac rehabilitation, physical activity, high-intensity interval training, medium-intensity continuous training, cardiorespiratory fitness, adherence to exercise

## Abstract

Exercise-based cardiac rehabilitation (ExCR) programs are essential for patients diagnosed with cardiac diseases. Studies have shown that they aid in the rehabilitation process and may even facilitate a return to previous cardiorespiratory fitness. Also, patients who enroll and follow such programs have shown a lower rate of complications and mortality in the long run. The results vary depending on the type of program followed and the degree of debilitation the disease has caused. Therefore, in order to obtain optimal results, it is ideal to tailor each ExCR program to the individual profile of each patient. At the moment, the two most studied and employed training types are medium-intensity continuous training (MICT) and high-intensity interval training (HIIT). For most of the time, MICT was the first-choice program for patients with cardiovascular disease. In recent years, however, more and more studies have pointed towards the benefits of HIIT and how it better aids patients in recovering their cardiovascular fitness. Generally, MICT is more suited for patients with a severe degradation in functional capacity and who require a higher degree of safety (e.g., elderly, with a high number of comorbidities). On the other hand, while HIIT is more demanding, it appears to offer better outcomes. Therefore, this review aimed to summarize information from different publications on both types of training regimens in ExCR and assess their utility in current clinical practice.

## 1. Introduction

Cardiac rehabilitation (CR) is a multifaceted intervention provided to patients with heart disease. It comprises elements of health education, physical activity, stress management, and counseling on cardiovascular risk reduction. There is growing evidence that it actually contributes to lowering mortality rates, illness incidence, and unplanned hospital visits while also enhancing exercise ability, quality of life, and psychological wellness. Therefore, CR is a procedure recommended by many international guidelines for patients diagnosed with cardiac diseases, especially coronary syndromes and heart failure [1,2,3,4,5].

Proper CR programs include exercise training, lifestyle changes, and psychological intervention. They are a cost-effective, class I A guideline recommendation that targets patients with cardiovascular disease [6].

Patients with a variety of heart conditions, including prior myocardial infarction (MI), coronary artery bypass surgery, valve replacement or repair surgery, percutaneous coronary procedures, chronic coronary syndrome, chronic heart failure (HF), or arterial hypertension, have been shown to benefit from such programs, especially exercise-based cardiac rehabilitation (ExCR) [6]. The main goals are to increase exercise capacity, preferably as close as possible to the pre-disease state, and to lower the overall cardiovascular risk of patients. This is also associated with lifestyle intervention, such as smoking cessation, improving lipid profile, controlling diabetes and blood pressure, and having a body mass index (BMI) in the normal range, while additional focus is placed on reducing stress, anxiety, and depression, which are often associated factors of such burdening diseases or surgical procedures [7,8,9].

Although mortality rates related to cardiovascular diseases have declined in European countries by more than 50% in the last 30 years, they are still the main culprit for disease related deaths, with over 1.5 million deaths in male patients and over 1.6 million deaths in females. In middle-income countries, mortality rates are 2.5 times higher than in high-income countries. Ischemic heart disease is the cardiovascular disease with the highest mortality, affecting approximately 171.4 males out of 100,000 and approximately 90.8 out of 100,000 in females. Stroke and heart failure are also more frequent in males than in females and in middle-income countries. Other conditions such as lower-extremity artery disease, atrial fibrillation, and degenerative mitral valve disease do not exhibit a significant difference between the two genders and are more frequently encountered in higher-income countries [10].

Each year, in the Federal Republic of Germany, about 10% of the government retirement fund allocation is directed to patients suffering from a type of cardiovascular disease, most frequently myocardial infarction or coronary artery disease [11]. In European registries, approximately one-third of the elderly are affected by acute coronary syndrome [12,13]. Besides the classic risk factors that are known to be associated with the development of coronary artery disease (e.g., smoking), other conditions that might impair a person’s ability to work and maintain independence include atrial fibrillation, which is the most common cardiac arrhythmia worldwide, and which affects approximately nine million people in Europe [14,15]. Since the financial impact is highly significant, secondary prevention through CR lessens the burden of cardiovascular disease on public healthcare [16].

The 2021 European Society of Cardiology (ESC) Guidelines on cardiovascular disease prevention in clinical practice mention that participation in a medically supervised, structured, comprehensive, multidisciplinary ExCR and prevention-oriented programs for patients after ischemic heart disease, with or without revascularization, and for patients with HF, especially HF with reduced ejection fraction (HFrEF), is a class I A recommendation [17].

There are several factors which have guided the writing of this review, which we encountered both in other published works and in our clinical practice in a cardiac rehabilitation center:

1. Not all cardiovascular patients are encouraged to enroll in or simply refuse to register for or follow a rehabilitation program;
2. There is still a general misbelief that a cardiovascular patient should rest and avoid any form of physical activity, despite evidence suggesting its benefits;
3. The choice of proper physical activity programs together with a specialized team in a rehabilitation center is vital for attaining the best outcomes, instead of choosing to perform different types of effort without proper instruction or understanding of functional capacity;
4. Although studies have been published on both types of programs, there are very few publications that actually compare the two.

Considering the factors mentioned above, this review focuses on the exercise component of rehabilitation program, and more precisely, on comparing the two main training concepts used nowadays: high-intensity interval training (HIIT) and medium-intensity continuous training (MICT), with the overall goal of highlighting their respective benefits for patients involved in ExCR programs.

For this purpose, we performed a search in the main databases containing medical publications. The following terms were used: cardiac rehabilitation, high-intensity interval training, resistance training, medium-intensity continuous training, exercise capacity, peak oxygen uptake, adherence to exercise, quality of life, adverse effects, and musculoskeletal injury.

Observational studies, systematic reviews, and meta-analyses were included. Studies describing other components of cardiac rehabilitation or including healthy subjects were excluded.

## 2. High-Intensity Interval Training

### Definitions

High-intensity interval training involves sets of relatively short periods of intense physical activity performed at a high workload, which corresponds to ≥90% of an individual’s maximum oxygen uptake (VO2 max), ≥90% of the minimal running speed required to achieve it [18], and >75% of maximal power output [18]. The exertion level is considered “hard” to “very hard”, corresponding to a rating of 6 or higher on the 10-point Borg scale of perceived exertion [19]. Each period of HIIT typically involves a maximum of several minutes of intense exercise, but it can also last just under 30 s, with the exact duration depending on the intensity. Multiple intense efforts are interspersed with rest periods lasting up to a few minutes or periods of lower exertion. Examples of HIIT exercises are listed in Table 1 [20].

Although it might instinctively seem more straightforward to evaluate the difficulty or impact of an exercise based on the heart rate achieved by the person involved, it is not consistently reliable if the equipment for determining VO2 max is not available [21,22]. This is mainly because heart rate can vary widely between individuals, especially those with heart conditions, meaning that their heart rate response to exercise may be exaggerated compared to healthy people [23]. Therefore, if exercise intensity is based only on maximal heart rate (HRmax), it could overestimate the appropriate training heart rate by up to 40% for certain individuals, suggesting that HR alone may not be an accurate guide for personalized exercise intensity [24,25].

## 3. Medium-Intensity Continuous Training

Moderate-intensity continuous training MICT is defined as physical activity that results in <80% peak HR or aerobic capacity and is performed over a more extended period as compared to HIIT types of activities [26].

The principles of MICT involve aerobic exercises for a prolonged duration at moderate intensity while maintaining the heart rate below 80% of the maximal value. This is compared to HIIT, where the heart rate surpasses 80% of the maximal value, but for short periods of time [27].

In 2023, D’Alleva et al. published a study evaluating the differences between a combined MICT and HIIT regimen and a solely MICT program on twenty-one obese adolescents. They underwent physical exercise sessions twice a day. The MICT protocol consisted of approximately 45 min at a heart rate corresponding to 40% of peak VO2. The intensity was adjusted using a treadmill by varying the speed and inclination. The combined protocol showed higher heart rate averages compared to the MICT group, but this was to be expected considering the HIIT component, which involves exercises at higher HR [28].

Also, following three weeks of MICT, all the participants showed increases in their peak VO2, as shown on subsequent exercise tests. Those who followed the MICT training showed higher amounts of oxidized lipids as compared to the group who followed the combined training regimen [28].

After three weeks of following these programs, the MICT group showed the highest decrease in body mass, with an average of approximately 8 kg of weight loss, of which an average of 4 kg was from fat mass. However, it should be noted that participants also showed a decrease in both fat-free mass and basal metabolic rate [28].

## 4. HIIT Versus MICT

### 4.1. Improvement in Cardiorespiratory Fitness

Perhaps the most important effect targeted through exercise training programs is improving the patient’s cardiorespiratory fitness. This concept denotes the ability of the cardiovascular and respiratory systems to deliver oxygen to the mitochondria in skeletal muscles, enabling energy production necessary for physical exertion. Thus, poor or unhealthy cardiorespiratory fitness levels strongly and independently predict cardiovascular disease and overall mortality among adults [29].

A systematic review and meta-analysis was conducted in 2017 to examine randomized controlled trials that describe cardiorespiratory fitness changes coming from high-intensity interval training compared to moderate-intensity continuous training [30].

Relevant databases were searched for studies published in English until July 2017, and in total, 16 studies with 953 participants (half of them assigned to HIIT and the other half to MICT) met the inclusion criteria [31,32,33,34,35,36,37,38,39,40,41,42,43,44,45,46]. The studies were grouped based on intervention duration: less than 6 weeks, more than 12 weeks, or somewhere in between. Meta-analyses were performed to analyze changes in peak VO2 and VO2 at the anaerobic threshold. Studies reporting results at two-time points were split into subgroups “a” and “b” [31,37,39,46]. Most studies showed significantly greater improvements (*p* < 0.05) in VO2 peak with HIIT compared to MICT, with only one study favoring MICT [33]. It was concluded that, regardless of intervention duration, HIIT is superior to MICT for improving peak VO2 [30].

The study’s secondary goal [30] was to look at the negative events of the randomized controlled trials that used high-intensity interval training compared to moderate-intensity continuous training in patients with heart conditions. It was noted that no study reported any deaths or cardiac problems during training that required hospitalization. Only one study pointed out three cardiac events in the MICT group: one that occurred the day after the training session, and two after the cardiac rehabilitation program was completed [39]. Although one study mentioned cases of angina that required participant withdrawal in both groups, more details were not provided [40]. Three (23%) of the thirteen studies that reported adverse events also mentioned additional negative effects of the HIIT intervention. These included common orthopedic injuries [38] due to the nature of the exercises, bronchitis [37], gastrointestinal disorders, and intermittent claudication [40]. The MICT branch was not clear of incidents either, with five studies (38%) documenting additional adverse events. These included knee injuries that ultimately required surgery [40], pericardial effusion [37], intestinal hemorrhage, bronchitis, lumbago, psychiatric exacerbations [40], musculoskeletal injury unrelated to training [41], and pain causing limited mobility of one’s limbs [42].

Later on, in 2020, results from a single-center randomized clinical trial (The FITR Heart Study) were published in the September issue of JAMA Cardiology. During this trial, participants engaged in four weeks of guided training as part of a cardiac rehabilitation program, followed by individual, unsupervised training at home and monitoring over a 12-month period—a significantly longer duration compared to the previous study. A total of 96 individuals diagnosed with coronary artery disease through angiography, aged between 18 and 80 years old, were recruited, with 93 of them receiving medical clearance for participation after a cardiopulmonary exercise test (CPET). Data collection took place over two and a half years, and analysis was conducted from December 2018 to August 2019. The patients were assigned to either a session of four sets of intense exercises with short pauses in between or to a longer session that included less strenuous exercises. In the first month, each participant had three sessions over the course of a week, two in the presence of a healthcare professional and one at home. After that, for the next 48 weeks, all three sessions took place at home. The primary focus was on the change in maximal oxygen uptake during the cardiopulmonary exercise test from baseline to 4 weeks. Additional assessments were carried out at 3, 6, and 12 months. The findings revealed that the HIIT group showed a 10% enhancement in VO2 peak after 4 weeks, compared to 4% in the MICT group. However, this beneficial impact of HIIT reached a plateau phase after that point, whereas MICT continued to improve the VO2 peak up to 7%. Thus, at the 12-month mark, neither of the two groups showed superiority [47].

At approximately the same time, another study was conducted (from July 2016 to March 2020) in the UK and published in February 2023 in the *European Journal of Preventive Cardiology.* Participants were divided into two groups for 8 weeks: one underwent twice-weekly HIIT (*n* = 187), while the other was assigned to MICT training (*n* = 195). HIIT involved ten sets of 1-min vigorous exercise intervals (>85% maximum capacity) with 1-min recovery periods in between. On the other hand, MICT consisted of less intense exercises (60–80% maximum capacity) over a longer period of time (minimum 20 min). The main goal was once again to observe how cardiorespiratory fitness changes after 8 weeks. This was assessed during CPET using a standard bicycle ramp protocol following established guidelines [48].

The results were consistent with those previously published, showing that after 8 weeks, the VO2 peak improved more significantly in the HIIT group compared to the MICT group, with only one adverse event potentially linked to HIIT [49].

### 4.2. Improvement in Vascular Function

While cardiovascular disease is known to be associated with rigid arteries and poor blood vessel function [50], regular physical activity has been demonstrated to have a direct beneficial impact on the vasculature. By exposing it to mechanical forces over a long period of time, it results in beneficial structural changes and functional adaptations [51]. Therefore, an optimal vascular function is associated with a reduced risk of cardiovascular events in the future and is indicated by flow-mediated dilation. This assessment shows an artery’s diameter change in response to increased blood flow [52].

Previous research has explored the comparison between HIIT and MICT in terms of improving vascular function, with HIIT showing greater enhancement in flow-mediated dilation compared to MICT. However, these findings were typically observed over extended training periods and in people who did not necessarily follow a CR program [53].

In contrast, the most recent paper addressing the issue of vascular improvement only in patients suffering from cardiovascular diseases was published in March 2022 in the *Scandinavian Journal of Medicine & Science in Sports* [54]. The goal was to see if the two training methods had a positive effect on the patient’s vasculature and, if so, which one of them displayed superiority. Its starting point was the FITR-Heart Study, but this time, the focus was mainly on the outcome of vascular function and associated variables (artery stiffness, blood pressure) [47].

In order to become eligible for the study, patients had to fulfill three criteria: first, a diagnosis of coronary artery disease based on the results of a coronarography; second, an age between 18 and 80 years; and lastly, the ability to participate in a cardiac rehabilitation program held at a private hospital [55]. During their first cardiac rehabilitation appointment, patients who fulfilled the requirements were invited to take part in the study. As previously mentioned, the study could be divided into three stages: (1) a 4-week program with two supervised and one home-based exercise sessions per week; (2) two more months of home-based training with at least three sessions per week; and (3) nine more months of home-based training with at least three sessions per week and no routine support [54]. Following the definitions of HIIT and MICT, the training programs were designed: one of them consisted of four intense sets (with a rate of perceived exertion between 15 and 18 on the Borg 6–20 scale [56]) interrupted by short pauses, while the other incorporated milder exercises. With the help of specialized equipment (SphygmoCor from Cardiex, Sydney, Australia), flow-mediated dilation was measured and analyzed according to standardized guidelines [46] in order to assess vascular function. The parameters evaluated according to the guidelines were: resting peripheral and central blood pressure, wave reflection, and arterial stiffness, including carotid to femoral pulse wave velocity [57].

The total number of eligible patients who agreed to participate in the study was 54. Subsequently, they were divided almost equally into two groups: HIIT and MICT. HIIT showed a more significant improvement in flow-mediated dilation than MICT after 4 weeks, but not 12 months. A possible explanation for this effect is that exercise of higher intensity leads to greater blood flow and shear stress, which increases the availability of nitric oxide [51,53]. After 4 weeks and over a span of 12 months, there were no notable differences in changes in pulse wave velocity and peripheral or central blood pressure between the two training methods. In summary, a 4-week HIIT program was better than MICT for enhancing vascular function, but not for improving arterial stiffness or blood pressure. Over the course of 12 months, both HIIT and MICT yielded similar results regarding changes in vascular function, blood pressure, and arterial stiffness [54].

### 4.3. Adherence to Exercise

Failure to adhere to and continue with regular exercise and physical activity is a significant factor preventing the field of exercise science from fully achieving its goal of enhancing public health worldwide. Research has claimed that HIIT is a viable and enduring type of exercise that may lead to greater long-term adherence than MICT [58]. Although research has concentrated on the effectiveness of HIIT for boosting cardiorespiratory fitness, especially in comparison to MICT, there has been less focus on adherence to each of these types of training [59].

To find out if this is true or not, Ekkekakis P. and Biddle S.J. [58] searched the main medical databases and identified eight trials that compared HIIT to MICT. All of them involved follow-up periods of at least 12 months. Their findings showed that, when left unsupervised, people who were initially advised to follow a HIIT program tend to lower the intensity of the recommended exercises. In the long run, HIIT groups did not demonstrate better adherence compared to others [53].

In 2021, another review of the literature was released [59], this time including 36 studies that were published in English up to September 2020. Out of the total, the concept of adherence was mentioned only in 13 studies [36,37,38,39,60,61,62,63,64,65,66,67,68,69,70,71,72], and it was determined based on the total number of training sessions attended and completed [59,60,61,62,63,64,65,66,67,68,69,70]. The percentage of completed sessions that defined adherence ranged from 66% to 100%. Most of these studies published their results as a comparison between the HIIT and the MICT programs [37,38,39,58,62,63,64,65,71,72,73,74,75,76,77,78,79,80,81,82,83,84]. For supervised programs, ten studies [37,38,39,40,41,42,44,73,78,82] compared session attendance between HIIT and MICT, all of which reported similar attendance between the two.

When it comes to observing adherence in the long-term, after the period of supervised training has finished, only two studies managed to stand out and report some results [37,58]. According to Moholdt et al., during 5 months of training at home, 64% of MICT participants and 52% of HIIT participants continued to train ≥3 times per week. In contrast to 4% of MICT participants who started more intense exercise, 35% of HIIT subjects stopped the intense training and switched to easier sessions instead [37]. On the other hand, The FITR-Heart Study discovered that, after the same time of home-based training, a greater percentage of MICT participants (38%) began higher-intensity exercise, whereas a smaller percentage of HIIT participants switched to a moderate-intensity regimen (24%). However, the proportion of HIIT participants who maintained their exercises ≥ three times per week in The FITR-Heart Study at 6 months (57%) and 12 months (53%) was consistent with the findings of Moholdt et al. Furthermore, the same adherence was observed in the MICT group at 6 and 12 months, respectively [58].

### 4.4. Quality of Life

A recent systematic review and meta-analysis, published in August 2023 [69], aimed to compare the effects of HIIT and MICT on the quality of life (QoL) and mental health (MH) of patients with cardiovascular disease. After searching the main medical databases, from their establishment until July 2023, a total of 5798 articles were carefully selected, of which only 22 were included in the meta-analysis according to the eligibility criteria [39,44,46,52,60,70,71,72,73,74,75,76,77,78,79,80,81,82,83,84,85,86]. The included studies originated from countries all over the world, among which Canada [46,75,85,86] and Norway [37,73,81,84] were the primary sources of articles. In the end, three conclusions could be drawn:

- HIIT and MICT had comparable impacts on quality of life for cardiovascular disease patients overall, but HIIT provided more significant physiological health benefits for CAD patients specifically;
- HIIT was more effective at reducing functional limitations originating from physical problems. It can also restore feelings of energy and enhance social adjustment capabilities;
- HIIT and MICT showed comparable effects in supporting mental health. However, HIIT increased exercise efficiency to a greater degree [70].

Therefore, the study found that high-intensity interval training should continue to be recommended for patients with cardiovascular diseases during their treatment and rehabilitation. This recommendation is based on the evidence that HIIT enhances these patients’ quality of life and mental health [69].

### 4.5. Safety

In apparently healthy, yet inactive people, participating in high-intensity aerobic exercise for the first time can substantially raise the chance of having a heart attack or, in rare cases, sudden cardiac death [87,88]. This elevated risk may be due to acute platelet aggregation and clot formation, increased thrombin and fibrin production, a spike in noradrenaline, adrenaline, and dopamine levels, or elevated shear forces on the endothelium that lead to atherosclerotic plaque instability and rupture. Subsequently, this information raised the question of whether HIIT can truly be recommended to people who are already suffering from a form of cardiovascular disease [89,90,91].

In a study including 25,420 cardiovascular patients, 20 major cardiac events were reported. Five happened during the cardiopulmonary exercise testing, while the others occurred during the actual training sessions. The frequency of events during exercise stress tests was reported as 1 per 8484, while during exercise training, it was 1 per 49,565 patient hours, with a cardiac arrest rate of 1.3 per 1,000,000 patient training hours [92]. These findings unequivocally demonstrate the high level of safety associated with the current cardiac rehabilitation program. Although HIIT shows a higher incidence of acute cardiac events compared to MICT, analyses suggest that the overall risk of cardiovascular events and musculoskeletal injuries with HIIT versus MICT remains low. However, the relatively small number of studied patients prevents drawing robust conclusions on the medical safety of HIIT [93].

Conversely, certain conditions such as valvulopathies, cardiac conduction disorders, and congenital heart disease raise concerns about the medical safety of HIIT [94]. Caution is also important when engaging in exercise training for individuals with nephropathy and retinopathy because an exaggerated vasopressor response could become dangerous. Special attention should also be given to patients suffering from diabetes complicated with peripheral and autonomic neuropathy, as balance issues or abnormal blood pressure/chronotropic response to exercise may appear. If foot deformities or preexistent wounds exist, they might make HIIT unsuitable, too. These factors contribute to why HIIT, while offering exercise variety, is not universally recommended for all cardiovascular patients as an alternative to MICT [95].

Therefore, based on the most recent and reliable information available, it is recommended that MICT continue to be the most practical and economical form of aerobic training for ongoing CR programs across all mentioned groups. HIIT may be recommended for certain individuals (e.g., those with stable coronary artery disease) to achieve particular treatment goals (such as improving maximal oxygen consumption) [94].

### 4.6. Impact on Left Ventricular Ejection Fraction

One important marker of HF is the left ventricular ejection fraction (LVEF), mainly because it directly influences the mortality rate [96]. Using eight databases, from their creation to July 5, 2023, a systematic search was conducted to analyze how the two types of exercise affect the patients’ LVEF. The results were displayed in a study that was published in February 2024 [97].

A total of thirteen randomized controlled trials were included in the study. They were carried out on individuals suffering from heart failure with reduced ejection fraction and included a total of 513 subjects, the majority (88%) being male. Approximately half of them (*n* = 262) underwent HIIT, whereas the rest successfully followed an MICT program. The average age observed among the participants was 63 years, with a BMI corresponding to the overweight category. The average LVEF at the beginning of the study was 34%, and the trials lasted from 3 and 5 weeks to 6 months. The primary modes of exercise adopted were bike riding and incline treadmill walking [72,81,84,98,99,100,101,102,103,104,105,106].

Out of the thirteen studies, eight of them [81,83,84,100,101,102,104,105] used the improvement of LVEF as the primary endpoint. The results showed that, in comparison to MICT, HIIT exhibited a notable enhancement in LVEF. However, due to the considerable variability observed, a sensitivity analysis was subsequently conducted. The analysis suggested that the study by Papathanasiou et al. might have played a role in this occurrence by focusing solely on a specific subset of patients (those with idiopathic dilated cardiomyopathy and cardiomyopathy of ischemic and hypertensive origin), instead of encompassing a larger spectrum of conditions. The exclusion of this study resulted in a significant decrease in heterogeneity [83].

## 5. Discussion

Cardiac rehabilitation aims to enhance both the function and structure of the heart in individuals suffering from cardiovascular disease, while also maximizing the patient’s overall well-being [104]. MICT has been the gold standard for exercise prescription for decades, until the HIIT regime surfaced [107].

Regarding cardiorespiratory fitness and vascular function improvement, results are clear: HIIT displays superiority in achieving its objectives compared to the usual MICT, but the maximum results are obtained in the short-term (after 4–8 weeks) [30,47,51,55]. The evaluation was continued after 3, 6, and 12 months. After one year, those differences became less significant, especially as only half of the participants managed to continue the program at home. Neither type of exercise showed superiority in keeping patients more engaged and less prone to quitting [37,38,39,40,41,42,44,59,60,108,109,110]. Moreover, new data that emerged in the past few months suggest that HIIT can improve the LVEF compared to MICT [97].

It is already well-known that physical activity can boost mood, concentration, alertness, and quality of life in general [107]. The literature indicates that both programs are effective, with HIIT demonstrating greater benefits. This may be attributed to participants perceiving more intensive physical training as more rewarding [69].

As cardiac rehabilitation targets people with cardiovascular diseases, the matters of safety and possible adverse events had to be addressed as well. After revising a series of published studies [92,93], the main conclusion was that the absolute risk for adverse cardiovascular events and musculoskeletal injury remains low. However, for certain populations, HIIT might not be the best option, meaning that the usual MICT remains the preferred exercise method for these patients [94].

Depending on the disease and the state of the patient, each of the programs has its own benefits which are summarized in Table 2.

The impact of HIIT on enhancing peak VO2 values in CR patients can be explained through its focus on oxidative metabolism. The successive effort-resting periods prolong the total oxidative predominance during sessions. HIIT also leads to a decrease in low-density lipoprotein (LDL)-cholesterol levels and an increase in high-density lipoprotein (HDL)-cholesterol levels. Higher adrenergic activity post-HIIT also decreases triglyceride transport and deposition in adipocytes and increases lipolytic activity [112].

However, it is important to mention that complete cardiac rehabilitation does not only involve physical exercises, but rather a complex team with an additional and equally important focus on treatment monitoring and optimization, as well as dietary and lifestyle intervention. Apart from issuing certain recommendations for the patients to follow, cardiac rehabiliation involves a patient education process through which they can better understand the impact of the disease and how they can influence the outcome [111].

A 2021 article published by Bozkurt et al. outlines the main steps of a proper rehabilitation program: baseline patient functional capacity, physical activity, tolerance assessment, individualized risk assessment for heart failure and comorbidities, individualized exercise prescription, monitored exercise, educational program, dietary and nutritional counseling, smoking cessation encouragement, psychological evaluation and intervention, monitoring of individual patient and overall program goals, comprehensive review of medications, including dosing and adherence, and long-term communication and interaction with appropriate physicians [111].

However, even when properly conducted, CR program outcomes are influenced by several factors such as age, gender, and BMI. Out of these, only BMI is a modifiable factor. In practical terms, this would translate into lower functional improvements in older, female, and obese patients, as addressed in a study by Bianci et al. [113].

In the case of older patients, several factors contribute to lower improvements during CR, including sarcopenia with age, a higher number of comorbidities, and possible drug interactions [113].

Female patients show a higher prevalence of spontaneous coronary dissection and myocardial infarction with no obstructive coronary atherosclerosis, while also presenting with atypical symptoms. These result in delayed diagnosis and treatment initiation. Furthermore, statistics show that male patients are more likely to join and adhere to a CR program [113].

Obese patients have shown radical improvements in their functional capacity when they managed to both lose weight and reach the normal parameters. Despite slower progress in these categories, CR has proven to be useful, with patients also showing better functional capacity, albeit not always reaching the maximum potential outcome [113].

Often overlooked is the psychological aspect of the cardiovascular patient. Unfortunately, depression is a risk factor which may lead to poorer outcomes. Treating it may result in a better prognosis during the CR. In coronary artery disease patients, effective communication is key and has been shown to significantly assist the recovery process. Selective serotonin uptake inhibitors are a useful therapy during cardiovascular rehabilitation [114].

Both of the programs are safe for patients suffering from cardiovascular disease, as the absolute risk for adverse cardiac events and musculoskeletal injuries appears to be low. However, some populations still require special attention, and further studies must be conducted before HIIT can be confirmed to be as safe for them as MICT.

The statements issued by the different studies incorporated into this review are important for practitioners in clinical rehabilitation centers, as they offer valuable insights into the two most applied training regimens for patients. By presenting the benefits of both and comparing them, patients could benefit from a better choice which could enhance their recovery process and lead to the best outcomes.

Several limitations should be acknowledged, including the heterogeneity of the studies, especially with regard to the number of patients. Unfortunately, many patients drop out from such exCR programs after phase II, meaning their performance cannot be followed in comparison to those who also undergo phase III on a long-term basis. Therefore, it is difficult to properly assess the evolution of every patient who follows such programs. Furthermore, there are many types of exercises involved in both MICT and HIIT regimens, which are also employed differently by each center. Future studies should focus on individually prescribed training programs and highlight the benefits enjoyed by those who followed them long-term compared to those who drop out.

## 6. Conclusions

While HIIT has clearly proved to offer better results when it comes to cardiorespiratory fitness and vascular function in the short term (4–8 weeks), this improvement becomes comparable with MICT after a year of cardiac rehabilitation. Neither of the two showed superiority in keeping patients engaged long-term. Quality of life and mental health are improved regardless of the training program used.

The general recommendations are to further encourage exCR programs and to promote their benefits. While HIIT offers slightly better results, MICT is safer and more suitable for patients with very low fitness levels, and they are both pillars of physical activity recovery in cardiovascular patients.

## Figures and Tables

**Table 1:** Examples of HIIT [20].

Number of Cycles	Low-Intensity	High-Intensity	Work-to-Recovery Ratio
3	15 s recovery between each activity	30 s each: push-ups squats triceps dips lunges (all types) jumping jacks crunches	2:1
4	20 s between every other activity; 1 min for each cycle	20 s each: push-ups with oblique knee (alternating) star jumps mountain climbers burpees high knees jumping lunges	2:1
4	4, 5 min low-intensity jog)	Running: 30 s maximal effort sprint	1:9
6	50 m slow—breaststroke	Swimming: 50 m sprint—freestyle	1:1
5	15 s rest between each distance; 1 min between each cycle	Basketball: sprint from baseline to a given point on the court and back	

**Table 2:** Effect of each type of program based on main disease [60,111].

Disease	Effect	MICT	HIIT
Heart failure	Similarity to lifestyle exercises	x	
	Time required		x
	Suitability for frail individuals or with very low fitness	x	
	CR team involvement		x
	Cardiometabolic benefit		x
	Fitness level obtained (VO2 improvement)		x
	Suitability for a broader range of patients	x	
	Safety	x	
Hypertension	Systolic blood pressure reduction	x	
	Diastolic blood pressure reduction	x	
Coronary syndrome	Short-term fitness improvement		x
	Long-term fitness improvement	x	x
	Adherence	x	
